# FOXO Transcriptional Factors and Long-Term Living

**DOI:** 10.1155/2017/3494289

**Published:** 2017-08-15

**Authors:** Ghulam Murtaza, Abida Kalsoom Khan, Rehana Rashid, Saiqa Muneer, Syed Muhammad Farid Hasan, Jianxin Chen

**Affiliations:** ^1^Beijing University of Chinese Medicine, Beisanhuan East Road, Beijing 100029, China; ^2^Department of Pharmacy, COMSATS Institute of Information Technology, Abbottabad, Pakistan; ^3^Institute of Automation, Chinese Academy of Sciences, Beijing, China; ^4^Department of Chemistry, COMSATS Institute of Information Technology, Abbottabad, Pakistan; ^5^Department of Pharmacy, University of Lahore, Lahore, Pakistan; ^6^Department of Pharmaceutics, Faculty of Pharmacy and Pharmaceutical Sciences, University of Karachi, Karachi, Pakistan

## Abstract

Several pathologies such as neurodegeneration and cancer are associated with aging, which is affected by many genetic and environmental factors. Healthy aging conceives human longevity, possibly due to carrying the defensive genes. For instance, FOXO (forkhead box O) genes determine human longevity. FOXO transcription factors are involved in the regulation of longevity phenomenon via insulin and insulin-like growth factor signaling. Only one FOXO gene (FOXO DAF-16) exists in invertebrates, while four FOXO genes, that is, FOXO1, FOXO3, FOXO4, and FOXO6 are found in mammals. These four transcription factors are involved in the multiple cellular pathways, which regulate growth, stress resistance, metabolism, cellular differentiation, and apoptosis in mammals. However, the accurate mode of longevity by FOXO factors is unclear until now. This article describes briefly the existing knowledge that is related to the role of FOXO factors in human longevity.

## 1. Introduction

Aging is related to the age-dependent impaired functioning of the cells, tissues, organs, and organ systems [1, 2]. This impairment leads to chronic pathologies including neurodegeneration, cardiovascular diseases, and cancer. Owing to these age-associated diseases, the researchers have always been interested in understanding the aging process and delaying the aging for human longevity [3, 4].

Healthy aging is a complex phenotype and an interplay of genetic and environmental factors such as food, exercise, and habits [5, 6]. However, rather than environmental factors, the contribution of genetic factors towards healthy aging is more significant. Thus, intensive studies have been done to investigate the genetic variants associated to human longevity.

**Figure 1:**
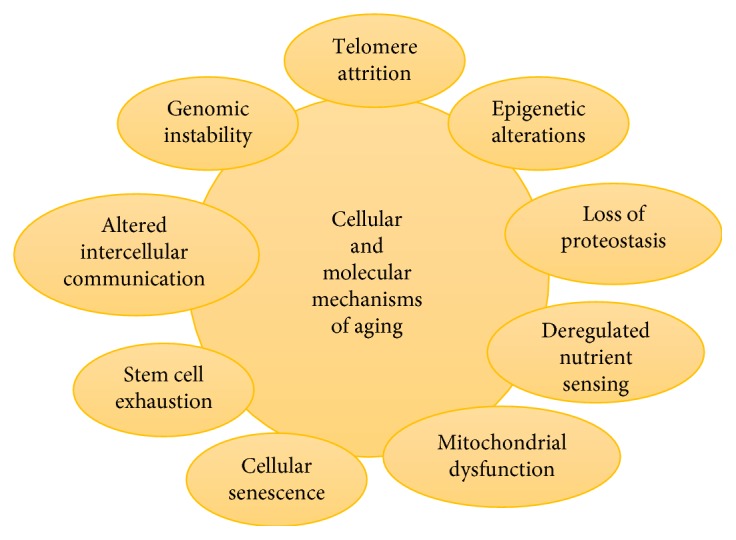
Various modes of aging.

Since health status affects the lifespan, the development of chronic diseases is delayed in the long-lived individuals [7–9]. These individuals could be the carriers of the defensive genes, which may be involved in body's defense against vulnerable moieties such as reactive oxygen species [10]. FOXO gene is one of the most crucial defensive genes that are known for ameliorating lifespan [4, 11–16]. Thus, aging can be considered an evolutionary process that is modulated by genetic programming and biochemical processes [17–20].  Figure 1 illustrates various modes of aging as studied in different organisms [18].

As observed in an animal study, the first ever documented mode of aging was deregulated nutrient sensing that involved the insulin and insulin-like growth factor- (IGF-) 1 signaling (IIS) pathway [21]. In IIS pathway, IGF-1 and insulin share and stimulate the same signaling pathways [22–24]. Thus, food that is an important environmental factor involved in IIS pathways must be cautiously used to achieve human longevity [23, 25, 26]. Other components of nutrient sensing pathways are the sirtuins, AMP (adenosine monophosphate) kinase, and the kinase mTOR (molecular target of rapamycin) [21].

## 2. FOXO Transcription Factors

The IIS pathway is highly influenced by the FOXO proteins [27, 28]. Forkhead box (FOX) transcription factor family was named after the Drosophila forkhead gene. The FOX family contains nineteen subfamilies of FOX genes, FOXA-FOXS, and is described by a highly conserved, winged-helix DNA-binding domain and the forkhead motif [29–32]. Other (O) subfamily of FOX, FOXO, is conserved from *Caenorhabditis elegans* (*C. elegans*) to mammals; only one FOXO gene exists in the invertebrates while mammals have 4 FOXO genes, FOXO1, FOXO3, FOXO4, and FOXO6 [29, 33, 34]. The alternate names FKHR, FKHRL1, and AFX have been used for FOXO1, FOXO3, and FOXO4, respectively [35]. FOXO1, FOXO4, and FOXO6 are overexpressed in the adipose, skeletal, and nervous tissues, respectively, while FOXO3 are excessively found in the spleen, stomach, intestine, kidney, and cardiac tissues [36]. These four genes are involved in the multiple cellular pathways, which regulate proliferation (FOXO1, FOXO3, and FOXO4), oxidative stress resistance (FOXO1 and FOXO3), metabolism (FOXO1 and FOXO3), cellular differentiation (FOXO3), inflammation (FOXO1, FOXO3, and FOXO4), aging (FOXO1, FOXO3, and FOXO4), and apoptosis (FOXO1, FOXO3, and FOXO4) in mammals [29–32, 37–40]. However, the involvement of these four genes in human longevity is still unrevealed.

FOXO proteins act as transcription activators and are suppressed by the IIS pathway [31, 37–40]. Concisely, P13K-AKT-mediated signaling pathway is activated by IGF-1 or insulin. It leads to the serine/threonine kinase AKT-induced phosphorylation of FOXO factors resulting in its nuclear exclusion and inhibition of FOXO-dependent transcription of target genes [41]. On the other hand, cellular stress leads to translocation of FOXO factors into the nucleus and activation of FOXO-dependent transcription. Besides phosphorylation, other posttranslational modifications including ubiquitination and methylation also influence the FOXO-dependent transcription [39, 40]. Thus, the FOXO posttranslational modifications lead to the aggregation of particular FOXO-dependent moieties that regulate various FOXO-dependent gene expressions [39, 42, 43]. In this article, various modes of human longevity involving FOXO transcription factors have been suggested.

## 3. Role of FOXO in Autophagy

**Figure 2:**
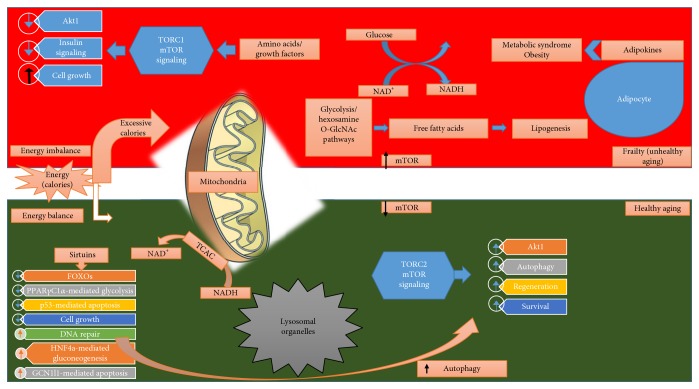
The crucial intracellular pathways targeted by FOXOs are presented here as modes of longevity effects of FOXOs. FOXOs are known to regulate translation of environment-induced stimuli into gene expression. FOXO-mediated longevity (especially through FOXO3) could be due to upregulated target genes pertained to apoptosis, cell cycle arrest, and resistance to stress leading to prevention of aging and age-associated diseases such as cancer and neurodegenerative diseases. The green part of the above figure illustrates cellular redox potential in mitochondria restoring NAD^+^. It results in the calorie restriction leading to various processes such as ameliorated autophagy, inhibited mTOR activity, and sirtuin-mediated activation of FOXOs giving rise to long-term living. While the red part of the above figure shows elevated levels of NADH due to excessive calorie intake resulting in lipogenesis, activated mTOR, excessive release of ROS, and suppressed autophagy leading to frailty. TCAC = tricarboxylic acid cycle; CAD = coronary artery disease; PPAR*γ*C1*α* = peroxisome proliferator-activated receptor-*γ* coactivator 1*α*; GCN1l1 = general control of amino acid synthesis 1-like 1; HNF4a = hepatocyte nuclear factor 4*α*; O-GlcNAc = O-linked N-acetylglucosamine.

The genes which mediate the intracellular clearance through autophagy and the ubiquitin-proteasome system are also regulated by FOXO factors [40, 44, 45]; thus, it can be suggested that FOXOs function as prolongevity factors. Starvation-induced defects in autophagy and the ubiquitin-proteasome system have been linked with the frailty and early aging (Figure 2) [46–48]. In addition, the genes which mediate the autophagy and mitophagy in muscle cells are also regulated by FOXO factors; it helps the tissues to adapt to starvation [49–51]. Moreover, Webb and Brunet [40] observed the activation of autophagy mechanisms by FOXO1 and FOXO3 in renal tubular cells, neurons, and cardiomyocytes. Since, FOXO factors play a role in proteasome-mediated degradation of short-lived cellular organelles and proteins, a suppressed proteasomal activity results in the aggregation of degraded proteins in the heart, liver, and muscle leading to aging [52, 53]. Additionally, the malfunctioned ubiquitin-proteasome system is a direct or indirect cause of various neurodegenerative diseases, for instance, Alzheimer's disease [40, 54, 55]. FOXO factors act on the neurodegenerative system via the upregulated ubiquitin ligases and by mediating the proteasome's composition [56–59]. However, the direct influence of proteostasis provoked by FOXO factors in mammals is not still disclosed.

## 4. Role of FOXO in Oxidative Stress

Antioxidant role of FOXO is its most crucial function. Since reactive oxygen species (ROS) produce conserved deteriorating effect on cells and induce aging, FOXOs could be used to influence aging by ameliorating the antioxidant potential of cells [60, 61]. ROS act as second messengers in various signaling pathways. An equilibrium in the production and degradation of ROS is necessary for normal cellular functioning, while imbalanced level of ROS results in abnormal functioning of the cells leading to various pathologies such as neurodegenerative diseases and cancer. Oxidative stress regulates FOXO factors, either through detection of cellular redox potential or modifying the upstream FOXO regulatory pathways [62, 63]. Normally, cellular detoxification keeps ROS level in normal range. An impaired cellular detoxification results in oxidative stress. Manganese superoxide dismutase (MnSOD), catalase, and GADD45 are major detoxification enzymes that are regulated by FOXOs [60, 64]. Hence, the inactivation of FOXOs result in the ROS built-up in the cells; it leads to various cellular abnormalities such as the compromised proliferation of normal stem cells but quick proliferation of transformed cells [65, 66].

## 5. Role of FOXO in Stem Cells

FOXO factors are known to be involved in stem cell biology. Aging is characterized with disequilibrium between removal and regeneration of cells in tissues, since the regeneration capability of adult stem cells is decreased with aging. Knockout hematopoietic stem cell (HSC) mice (mice with FOXO1/3/4-deficient hematopoietic stem cells) showed apoptosis of HSCs as well as termination of repopulation of HSCs. Likewise, FOXO3-deficient mice illustrated the reduced potential of regeneration of cells [67]. The deletion of FOXO factor could lead to exhaustion of the respective stem cell pool [68]. Surprisingly, the HSC compartment was restored in FOXO-deficient mice treated with an antioxidant N-acetylcysteine, proposing that stem cells are disturbed by accumulation of ROS. This finding supports the hypothesis that oxidative stress contribute primarily to aging, while malfunctioned adult stem cells have secondary significance in this context [69]. Besides playing a role in adult stem cells, FOX factors mediate the expression of OCT4 and SOX2 factors associated with stemness. Similarly, FOXO1 factors are pluripotent for human embryonic stem cells (ESC), and the ortholog FOXO1 plays similar role in mouse ESCs [70].

## 6. FOXO Factors and Long-Term Living

This review article narrates a summary of the prevalent knowledge that is associated to the role of FOXO factors in extending human lifespan. Until now, no study describes the exact mode of action of FOXOs in human aging. However, some studies on various populations narrate the possible role of FOXO factors in human longevity.

The older the age, larger is the contribution of genetics in lifespan stating genetics as a function of human longevity. Thus, genetics is the basic parameter that discriminates the average-lived population from the centenarians [1, 2, 71, 72]. Thus, the centenarians are rich in specific alleles, which possibly represent the genes contributing to human longevity. These genes are therefore extensively being investigated in current years.

First study of this type narrated the association of human longevity with FOXO3A [73]. This study was performed on 4 genes named as FOXO1A, FOXO3A, SIRT1, and COQ7 and one SNP, named as rs2764264, in long-lived American males of Japanese origin. Only FOXO3A and rs2764264 were observed to have association with human longevity among the studied genes and SNPs [73]. The incidence rate of age-associated pathologies such as cancer and neurodegenerative and cardiovascular diseases in these individuals was also lower than control group. The control group was eleven years younger than test. The significantly lower level of insulin in the control group was also due to the same allele [73].

Subsequently, same association was found in male centenarians from Italy [11], Germany [12], and Denmark [13]. In an Italian study, rs2802288 exhibited the maximum allelic relationship-minor allele frequency. All three studies described the significant association between FOXO3 polymorphism and human longevity. While, the Danish study proposed four new single nucleotide polymorphisms (SNPs) (named as rs9400239, rs2764264, rs479744, and rs13217795) associated with human longevity [13].

In other study, two SNPs from FOXO1A (rs2755209 and rs2755213) and three from FOXO3A (rs4946936, rs2802292, and rs2253310) were analyzed in Chinese centenarians [74]. All the six SNPs were positively and gender-independently linked with long-term survival, except two SNPs from FOXO1A that were negatively linked with longevity in female subjects [74]. The conclusion of study states that there is strong association between FOXO1A and long lifespan in females showing the influence of gender in genetic association to human longevity [74]. Another study in Chinese population reported the gender involvement in the impact of FOXO1A and FOXO3A SNPs independently on human longevity [75–77]. Similar finding showing the importance of genetics in the IIS pathway in long-lived Jews and people of Italy, Japan, and Netherlands has also been reported [78–81]. In addition, human longevity has also been found to be associated with other five FOXO3A SNPs (named as rs2802288, rs2802292, rs1935949, rs13217795, and rs2764264) [15]. Among these five SNPs, rs2802292 and rs2764264 polymorphisms were observed in males only.

The combined effect of FOXO3A and APOE on long-term living has also been reported [2, 16, 82]. Additionally, an increase in the activity of daily life and decrease in the risk of bone fracture in individuals with FOXO3A SNPs were found in long-lived Danish individuals [14]. Conclusively, there is remarkable association between human longevity and FOXO3A SNPs as evident from above cited studies conducted in various populations. However, the translation of FOXO3 gene sequences into phenotypic features that facilitate a long-term living is still unrevealed. Moreover, rather than associating with known SNPs, FOXO3A alleles related to long-term living act as introns [2, 72, 83]. It proposes that these SNPs are expected to influence FOXO3A without affecting protein functionality.

## 7. Prediction of Mechanisms of FOXO in Long-Term Living

Network pharmacology is a multidisciplinary field that integrates different scientific concepts such as systems biology, cheminformatics, and bioinformatics to explore various novel bioactivities from network-based analysis. For instance, network pharmacology helps us to study gene characteristics and its functions [84], identify therapeutic targets, and explore the mode of action of various drugs [85]. Thus, network pharmacology is used here to predict the possible modes of action of FOXOs in human longevity.

STITCH 4.0 database (http://stitch.embl.de/) [86] has been used to retrieve targets (confidence score > 0.4) of FOXO1, FOXO3, FOXO4, and FOXO6 in the form of protein-protein network (Supplementary data, Figure 3, Table 1 available online at https://doi.org/10.1155/2017/3494289). These protein targets were fetched into Cytoscape, and protein-protein interaction network was constructed to visualize the functionality-associated genes. The functional enrichment analysis was conducted by using the gene ontology terms (GO terms) for annotation of the biological functions of FOXO-related targets. Subsequently, Cytoscape plug-in ClueGO [87] was utilized to analyze FOXO-mediated biological process term (BP term) to explore the biological importance of the specific targets linked to FOXO1, FOXO3, and FOXO4. Overall, the significant enrichment of 14, 16, and 7 GO terms was achieved for FOXO1, FOXO3, and FOXO4 (Supplementary data, Figure 4, Table 2). The effect of FOXOs on these BPs has been reported by some investigators. FOXOs are mainly involved in the regulation of metabolism, regulation of reactive species, and regulation of cell cycle arrest and apoptosis. FOXO1 regulates adipogenesis, gluconeogenesis, and glycogenolysis. Mechanistically, the unphosphorylated FOXO1 binds to the insulin response sequence present in the promoter region of G6P (glucose-6 phosphatase) in the nucleus [88]. It leads to the accelerated transcription resulting in the enhanced production of glucose in the liver. After Akt-mediated phosphorylation, FOXO1 is transferred to the cytoplasm and undergoes ubiquitination and degradation. It leads to the decreased production of glucose in the liver via decreased transcription of G6P leading to the decreased rate of gluconeogenesis and glycogenolysis [89]. Adipogenesis is negatively regulated by FOXO1 through its binding to the promoter region of PPARG (peroxisome proliferator-activated receptor gamma) and inhibiting its transcription [90]. It results in the FOXO1-mediated inhibition of adipogenesis [91]. The initiation of adipogenesis requires the increased levels of PPARG [92, 93]. Moreover, FOXO1 functions as an association between transcription and insulin-mediated metabolic control; thus, FOXO1 is a promising genetic target to manage type 2 diabetes.

FOXO3 probably induces apoptosis either upregulating the genes needed for cell death [94] or downregulating the antiapoptotic factors [95]. In addition, FOXO3 has been found to regulate Notch signaling pathway during the regeneration of muscle stem cells [96]. Moreover, antioxidants are thought to be upregulated by FOXO3 to protect human health from oxidative stress. Additionally, FOXO3 is documented to suppress tumour [97]. Thus, tumour development may occur if FOXO3 is deregulated. Most importantly, FOXO3 are described to play a role in long-term living [12].

FOXO4 is involved in the regulation of various pathways associated to apoptosis, longevity, cell cycle, oxidative stress, and insulin signaling. FOXO4 are associated with longevity through IIS pathway [98, 99]. Finally, mutation-triggered Akt phosphorylation results in the inactivated FOXO4 [100]. It deregulates cell cycle and activates kinase inhibitor involved in cell cycle [101, 102]. It leads to the prevention of tumour progress into G1. These biological processes make us better understand the modes of action of FOXOs.

## 8. Conclusions

In current years, the rigorous research attention has been focused on the role of FOXO transcription factors in human longevity. In different animal models, numerous studies have been conducted to investigate the signaling pathways involved in the regulation of the FOXO factors. Moreover, the effect of FOXO-mediated processes on the cellular, tissue, or organism level functions has also been discussed. As a result, a pleiotropic nature of FOXOs' effect on longevity is established, since FOXOs participate in a number of cellular functions, including growth, stress resistance, metabolism, cellular differentiation, and apoptosis. From the above discussion, numerous strategies for future research can be predicted. For instance, the triggering of FOXO-mediated processes in the tissues with metabolically different features can be valuable to explore the mechanism of FOXO-mediated longevity. In addition, the human FOXO sequence variations and their effect on the resulting proteins should be studied, the possible findings can also reveal the underlying mechanisms of FOXO-induced health aging. The delay in age-related pathologies including cancer and neurodegenerative diseases and living long life depends on the control of morbidity. It is therefore an exciting area of study to investigate the antiaging compounds; however, their testing in clinical setup would need age markers to assess aging rate. Owing to the potential effect of FOXOs on health issues, the future therapies could be based on the FOXOs.

## Supplementary Material

Figure 3. Confidence view of the protein networks of FOXO1, FOXO3, FOXO4 and FOXO6. Thick lines indicate stronger associations. Grey and green lines represent the protein-protein. SIRT1 - Sirtuin 1; AKT1 - v-akt murine thymoma viral oncogene homolog 1; AKT2 - v-akt murine thymoma viral oncogene homolog 2; AKT3 - v-akt murine thymoma viral oncogene homolog 3; BCL2L11 - BCL2-like 11; CDK2 - Cyclin-dependent kinase 2; CDKN1A - Cyclin-dependent kinase inhibitor 1A; CDKN1B - Cyclin-dependent kinase inhibitor 1B; CREBBP - CREB binding protein; CTNNB1 - Catenin (cadherin-associated protein), beta 1; FHL2 - Four and a half LIM domains 2; EP300 - E1A binding protein p300; GADD45A - Growth arrest and DNA-damage-inducible, alpha; IKBKB - Inhibitor of kappa light polypeptide gene enhancer in B-cells; INS – Insulin; MAPK8 - Mitogen-activated protein kinase 8; SGK1 - Serum/glucocorticoid regulated kinase 1 (526 aa); SGK2 - Serum/glucocorticoid regulated kinase 2; SGK3 - Serum/glucocorticoid regulated kinase family, member 3; SMAD2 - SMAD family member 2; SMAD3 - SMAD family member 3; SMAD4 - SMAD family member 4; STK4 - Serine/threonine kinase 4; PRKAA2 - Protein kinase, AMP-activated, alpha 2 catalytic subunit; YWHAZ - Tyrosine 3-monooxygenase/tryptophan 5-monooxygenase activation protein, zeta polypeptide and USP7 - Ubiquitin specific peptidase 7. Table 1. Protein-protein network stats. Figure 4. Organic layout algorithm of functionally grouped networks produced through ClueGO analysis to predict the potential targets of FOXO1, FOXO3, FOXO4 and FOXO6. Each group comprises of the most significant terms only. The overlapped groups indicates their functional likeness. Table 2. GO terms and their associated genes.
