# Supplementary material for: FOXO Transcriptional Factors and Long-Term Living
Source: Oxid Med Cell Longev. 2017 Aug 15;2017:3494289. doi: 10.1155/2017/3494289 (PMC5574317; doi:10.1155/2017/3494289)
Supplement: Supplementary file 1 — Figure 3. Confidence view of the protein networks of FOXO1, FOXO3, FOXO4 and FOXO6. Thick lines indicate stronger associations. Grey and green lines represent the protein-protein. SIRT1 - Sirtuin 1; AKT1 - v-akt murine thymoma viral oncogene homolog 1; AKT2 - v-akt murine thymoma viral oncogene homolog 2; AKT3 - v-akt murine thymoma viral oncogene homolog 3; BCL2L11 - BCL2-like 11; CDK2 - Cyclin-dependent kinase 2; CDKN1A - Cyclin-dependent kinase inhibitor 1A; CDKN1B - Cyclin-dependent kinase inhibitor 1B; CREBBP - CREB binding protein; CTNNB1 - Catenin (cadherin-associated protein), beta 1; FHL2 - Four and a half LIM domains 2; EP300 - E1A binding protein p300; GADD45A - Growth arrest and DNA-damage-inducible, alpha; IKBKB - Inhibitor of kappa light polypeptide gene enhancer in B-cells; INS – Insulin; MAPK8 - Mitogen-activated protein kinase 8; SGK1 - Serum/glucocorticoid regulated kinase 1 (526 aa); SGK2 - Serum/glucocorticoid regulated kinase 2; SGK3 - Serum/glucocorticoid regulated kinase family, member 3; SMAD2 - SMAD family member 2; SMAD3 - SMAD family member 3; SMAD4 - SMAD family member 4; STK4 - Serine/threonine kinase 4; PRKAA2 - Protein kinase, AMP-activated, alpha 2 catalytic subunit; YWHAZ - Tyrosine 3-monooxygenase/tryptophan 5-monooxygenase activation protein, zeta polypeptide and USP7 - Ubiquitin specific peptidase 7. Table 1. Protein-protein network stats. Figure 4. Organic layout algorithm of functionally grouped networks produced through ClueGO analysis to predict the potential targets of FOXO1, FOXO3, FOXO4 and FOXO6. Each group comprises of the most significant terms only. The overlapped groups indicates their functional likeness. Table 2. GO terms and their associated genes. [file 3494289.f1.docx]

**Supplementary data**

| 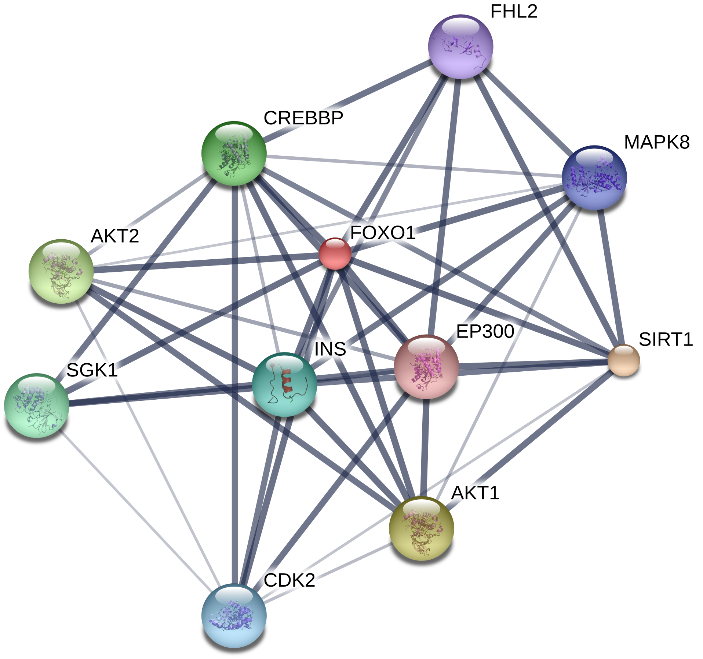 | 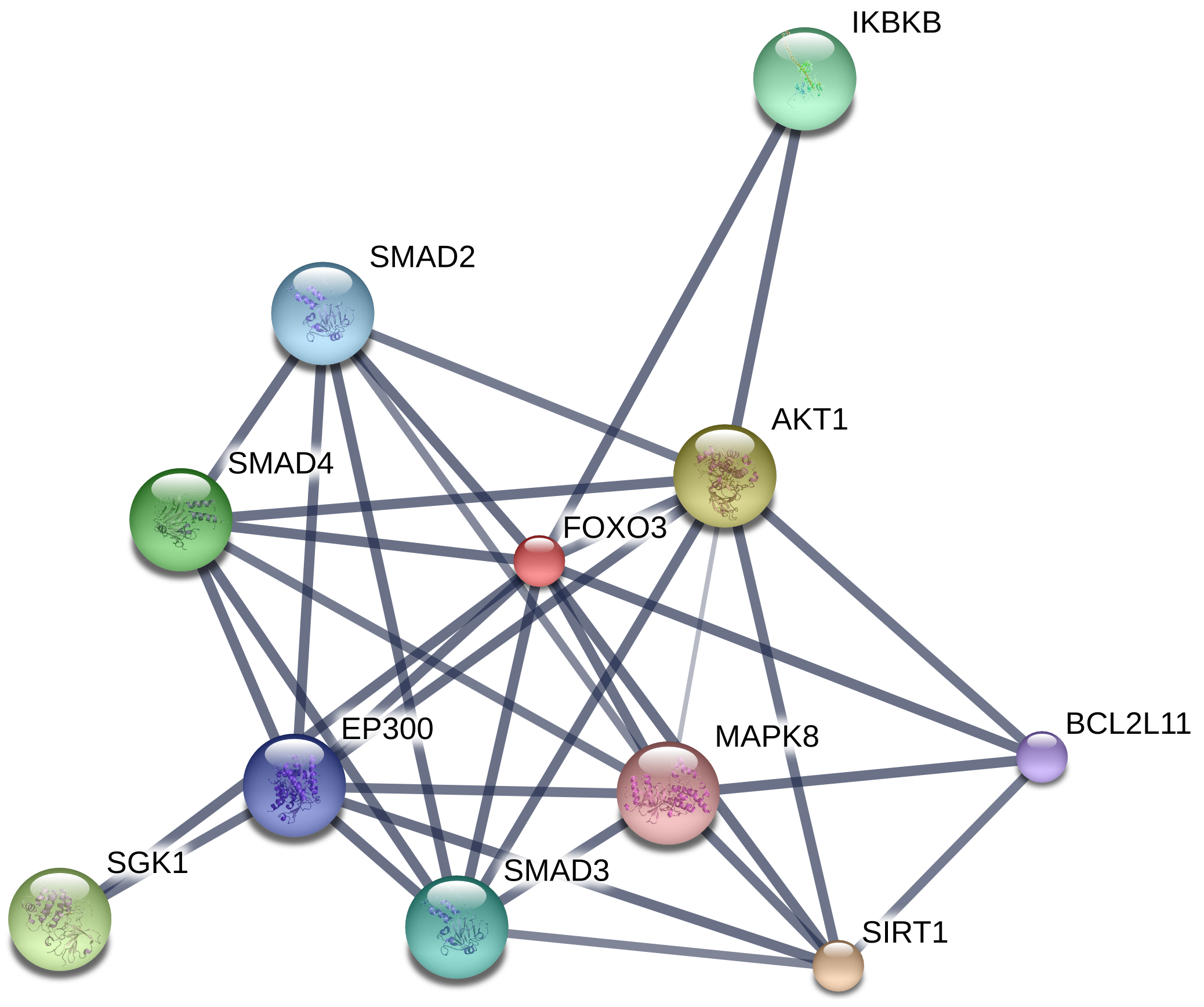 |
| --- | --- |
| FOXO1 | FOXO3 |
| 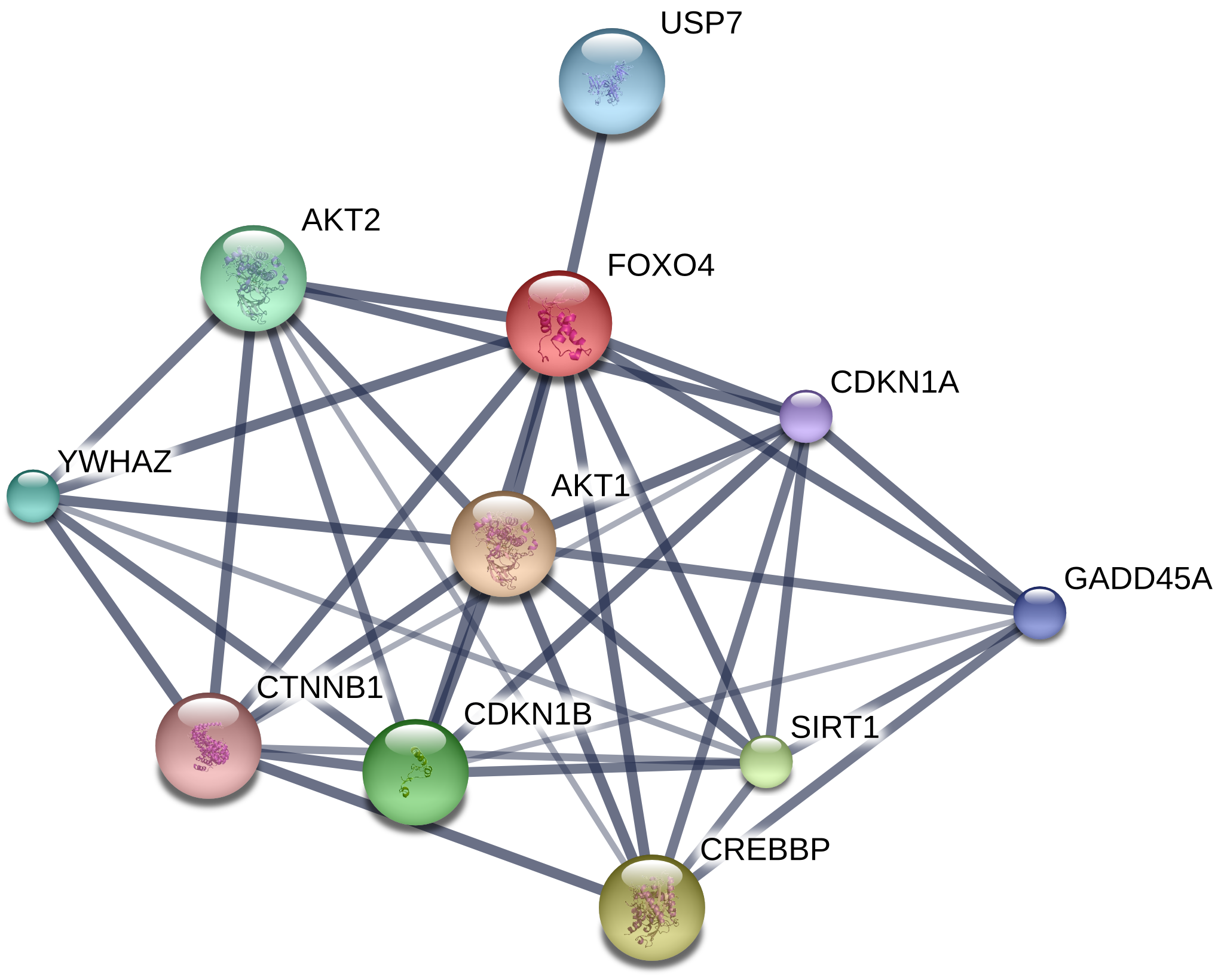 | 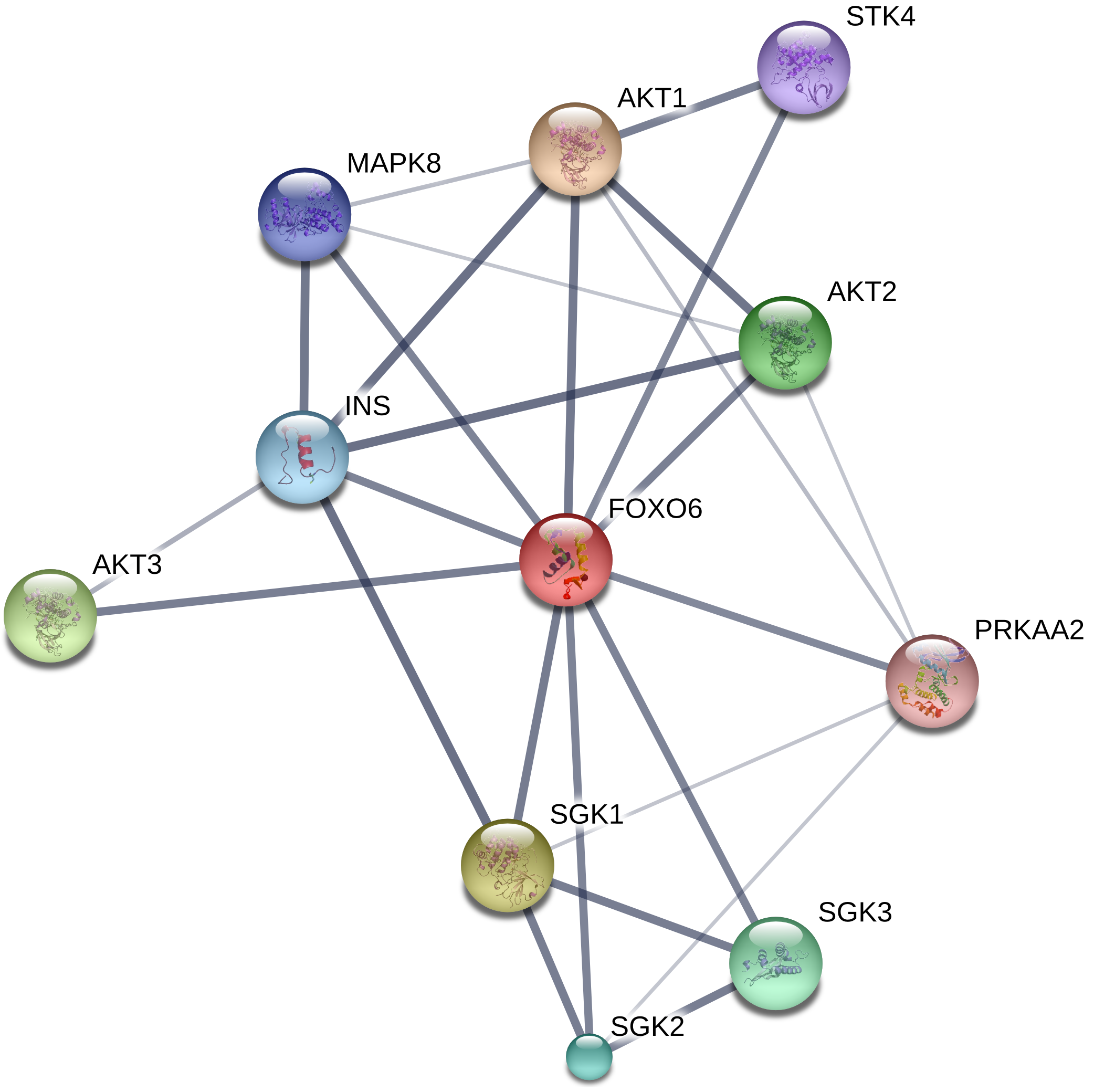 |
| FOXO4 | FOXO6 |

Figure 3. Confidence view of the protein networks of FOXO1, FOXO3, FOXO4 and FOXO6. Thick lines indicate stronger associations. Grey and green lines represent the protein-protein. SIRT1 - Sirtuin 1; AKT1 - v-akt murine thymoma viral oncogene homolog 1; AKT2 - v-akt murine thymoma viral oncogene homolog 2; AKT3 - v-akt murine thymoma viral oncogene homolog 3; BCL2L11 - BCL2-like 11; CDK2 - Cyclin-dependent kinase 2; CDKN1A - Cyclin-dependent kinase inhibitor 1A; CDKN1B - Cyclin-dependent kinase inhibitor 1B; CREBBP - CREB binding protein; CTNNB1 - Catenin (cadherin-associated protein), beta 1; FHL2 - Four and a half LIM domains 2; EP300 - E1A binding protein p300; GADD45A - Growth arrest and DNA-damage-inducible, alpha; IKBKB - Inhibitor of kappa light polypeptide gene enhancer in B-cells; INS – Insulin; MAPK8 - Mitogen-activated protein kinase 8; SGK1 - Serum/glucocorticoid regulated kinase 1 (526 aa); SGK2 - Serum/glucocorticoid regulated kinase 2; SGK3 - Serum/glucocorticoid regulated kinase family, member 3; SMAD2 - SMAD family member 2; SMAD3 - SMAD family member 3; SMAD4 - SMAD family member 4; STK4 - Serine/threonine kinase 4; PRKAA2 - Protein kinase, AMP-activated, alpha 2 catalytic subunit; YWHAZ - Tyrosine 3-monooxygenase/tryptophan 5-monooxygenase activation protein, zeta polypeptide and USP7 - Ubiquitin specific peptidase 7.

Table 1. Protein-protein network stats

| Protein-protein network parameters | FOXO1 | FOXO3 | FOXO4 | FOXO6 |
| --- | --- | --- | --- | --- |
| Number of nodes | 11 | 11 | 11 | 11 |
| Number of edges | 45 | 34 | 39 | 26 |
| Average node degree | 8.18 | 6.18 | 7.09 | 4.73 |
| Clustering coefficient | 0.876 | 0.859 | 0.853 | 0.757 |
| Expected number of edges | 24 | 19 | 19 | 17 |
| PPI enrichment p-value | 0.0000802 | 0.00138 | 0.0000451 | 0.0252 |

| 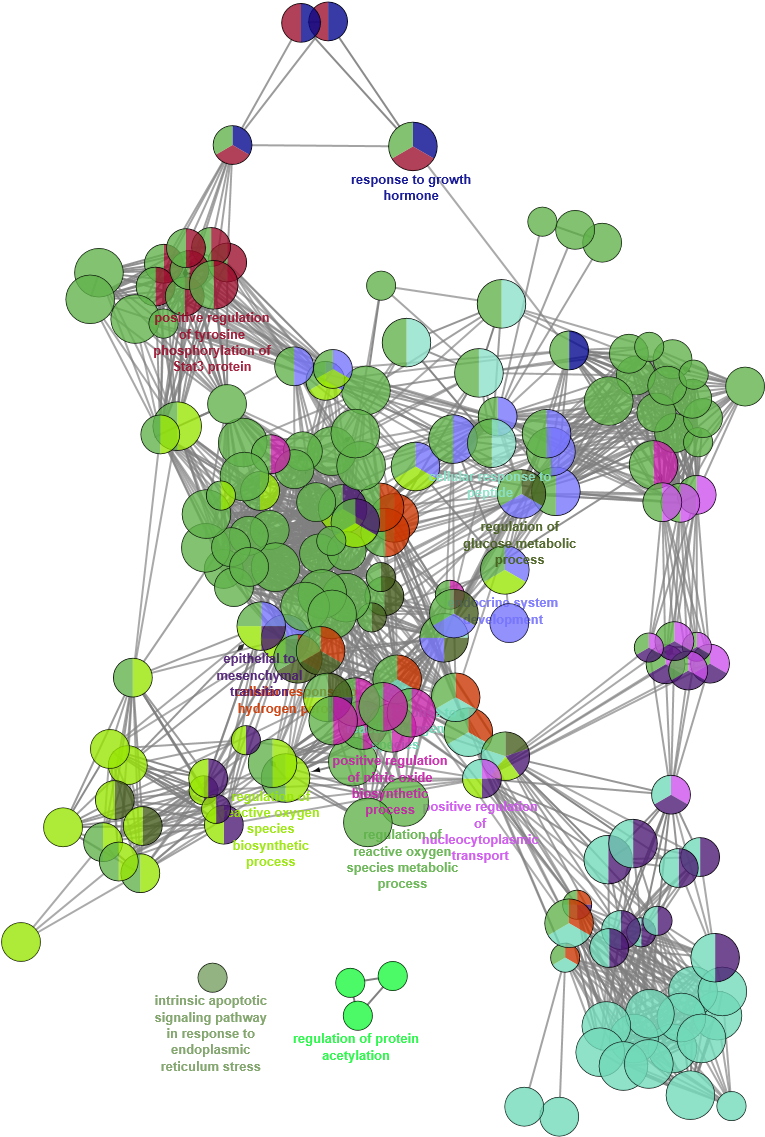 |
| --- |
| FOXO1 |
| 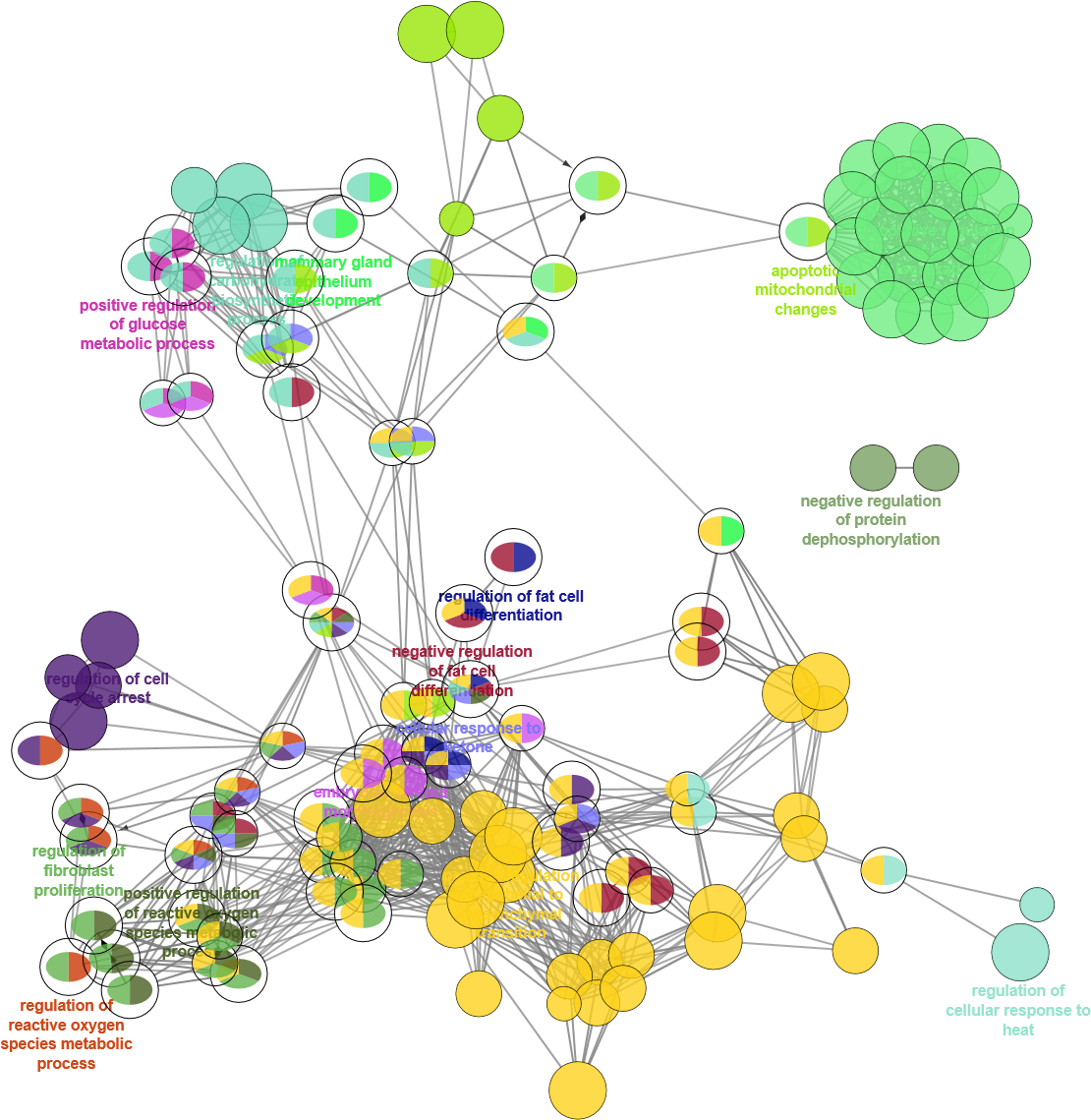 |
| FOXO3 |
| 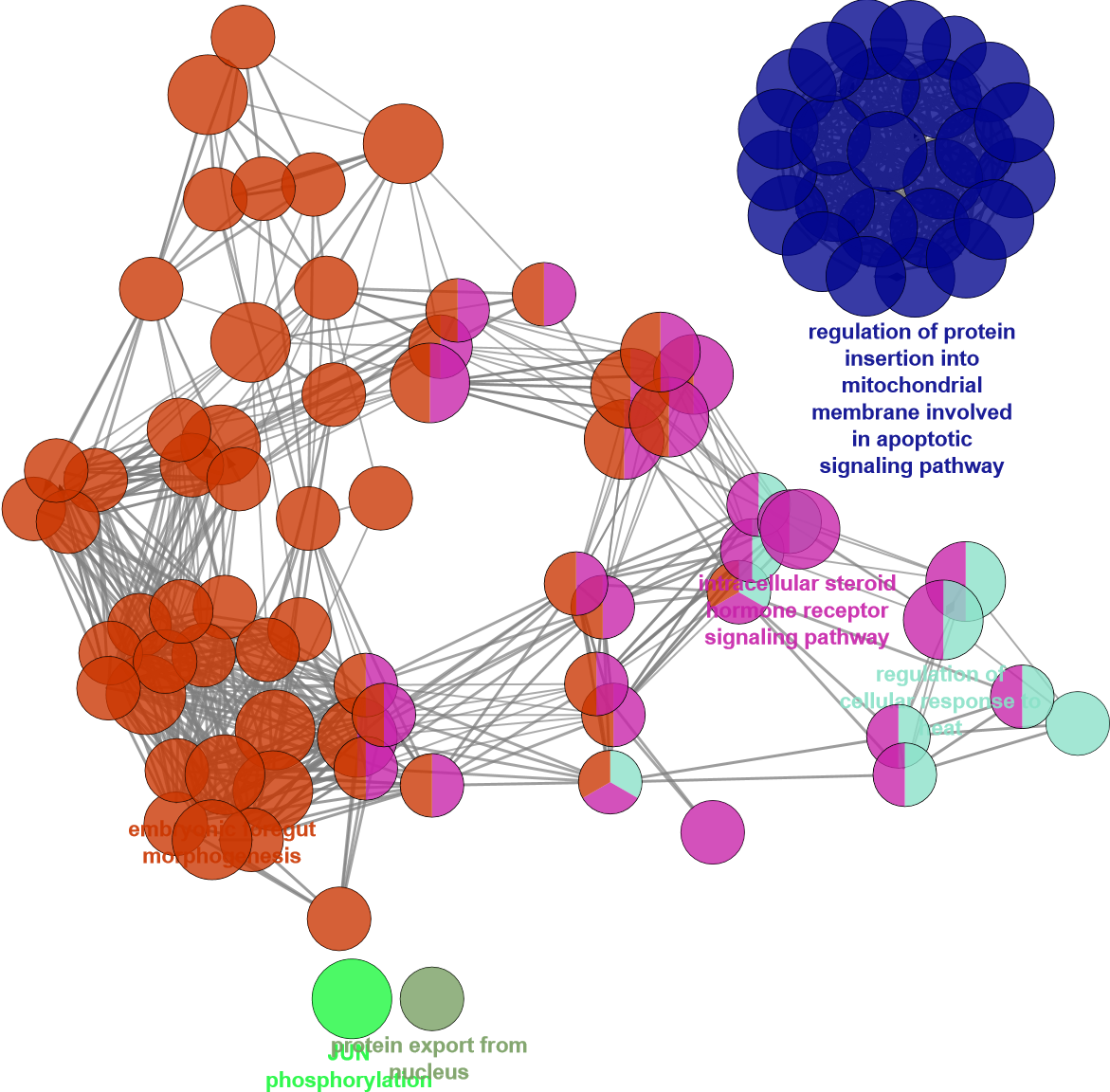 |
| FOXO4 |

Figure 4. Organic layout algorithm of functionally grouped networks produced through ClueGO analysis to predict the potential targets of FOXO1, FOXO3, FOXO4 and FOXO6. Each group comprises of the most significant terms only. The overlapped groups indicates their functional likeness.

Table 2. GO terms and their associated genes

| GOID | GO Term | Associated Genes Found |
| --- | --- | --- |
| FOXO1 | | |
| GO:0070059 | Intrinsic apoptotic signaling pathway in response to endoplasmic reticulum stress | [BCL2L11, GSK3B, SIRT1] |
| GO:1901983 | Regulation of protein acetylation | [MAPK3, SIRT1, SMAD4] |
| GO:1901653 | Cellular response to peptide | [AGTR1, AKT1, AKT2, FOXO1, FOXO3, FOXO4, GAB1, GRB2, HNF4A, IL6, KLF2, MAPK3, SIRT1, SOS1, STAT3, YWHAG] |
| GO:0060416 | Response to growth hormone | [AKT1, HNF4A, MAPK3, STAT3] |
| GO:0045429 | Positive regulation of nitric oxide biosynthetic process | [AKT1, ESR1, IL6, KLF2, SMAD3] |
| GO:0070301 | Cellular response to hydrogen peroxide | [CDK1, FOXO1, FOXO3, IL6, KLF2, SIRT1, SOS1] |
| GO:0046824 | Positive regulation of nucleocytoplasmic transport | [CDK1, GSK3B, IL6, SMAD3, SMAD4] |
| GO:0042517 | Positive regulation of tyrosine phosphorylation of Stat3 protein | [IL6, IL6R, IL6ST, STAT3] |
| GO:0010906 | regulation of glucose metabolic process | [AKT1, AKT2, FOXA2, FOXO1, GSK3B, IL6, SIRT1] |
| GO:0035270 | Endocrine system development | [AKT1, FOXA2, FOXO1, GSK3B, HOXA5, IL6, IL6R, MAPK3, SMAD3] |
| GO:0001837 | Epithelial to mesenchymal transition | [FOXA2, GSK3B, IL6, SMAD2, SMAD3, SMAD4, SOS1] |
| GO:1903426 | Regulation of reactive oxygen species biosynthetic process | [AKT1, ESR1, FOXO3, IL6, KLF2, SMAD3, STAT3] |
| GO:0034614 | Cellular response to reactive oxygen species | [CDK1, CDK2, FOXO1, FOXO3, IL6, KLF2, SIRT1, SOS1] |
| GO:2000377 | Regulation of reactive oxygen species metabolic process | [AGTR1, AKT1, CDKN1A, ESR1, FOXO1, FOXO3, GRB2, IL6, KLF2, SIRT2, SMAD3, STAT3] |
| FOXO3 | | |
| GO:0035308 | Negative regulation of protein dephosphorylation | [IKBKB, YWHAB, YWHAE] |
| GO:0061180 | Mammary gland epithelium development | [AKT1, AKT2, CEBPB, ESR1, MST1, STK4, TGFB1] |
| GO:1900034 | Regulation of cellular response to heat | [CREBBP, EP300, FKBP4, MAPK3, SIRT1, YWHAE] |
| GO:0045598 | Regulation of fat cell differentiation | [AKT1, CEBPB, DDIT3, FOXO1, SIRT1, SMAD3, SOD2, STK4, TGFB1] |
| GO:0010907 | Positive regulation of glucose metabolic process | [AKT1, AKT2, FOXO1, IRS2, KAT2B] |
| GO:2000377 | Regulation of reactive oxygen species metabolic process | [AKT1, CDKN1A, ESR1, FOXM1, FOXO1, FOXO3, SIRT3, SMAD3, SOD2, TGFB1, TP53] |
| GO:0048617 | embryonic foregut morphogenesis | [CTNNB1, SMAD2, SMAD3] |
| GO:0045598 | Regulation of fat cell differentiation | [AKT1, CEBPB, DDIT3, FOXO1, SIRT1, SMAD3, SOD2, STK4, TGFB1] |
| GO:2000379 | Positive regulation of reactive oxygen species metabolic process | [AKT1, CDKN1A, ESR1, FOXO3, SMAD3, SOD2, TGFB1, TP53] |
| GO:1901655 | Cellular response to ketone | [AKT1, FOXO1, FOXO3, MSTN, SIRT1, TGFB1] |
| GO:0071156 | Regulation of cell cycle arrest | [AKT2, CDKN1A, EP300, FOXM1, FOXO4, SFN, TGFB1, TP53] |
| GO:0008637 | Apoptotic mitochondrial changes | [AKT1, BCL2L11, SFN, SOD2, TNFSF10, TP53, YWHAB, YWHAE, YWHAG, YWHAH, YWHAQ, YWHAZ] |
| GO:0043255 | Regulation of carbohydrate biosynthetic process | [AKT1, AKT2, FOXO1, IRS2, KAT2B, MST1, STK4, TGFB1] |
| GO:0048145 | Regulation of fibroblast proliferation | [CDK6, CDKN1A, CTNNB1, ESR1, SOD2, TGFB1, TP53] |
| GO:1905477 | Positive regulation of protein localization to membrane | [AKT1, AKT2, BCL2L11, SFN, TGFB1, TP53, YWHAB, YWHAE, YWHAG, YWHAH, YWHAQ, YWHAZ] |
| GO:0010718 | Positive regulation of epithelial to mesenchymal transition | [CTNNB1, SMAD2, SMAD3, SMAD4, TGFB1] |
| FOXO4 | | |
| GO:0006611 | Protein export from nucleus | [RAN, SFN, XPO1] |
| GO:0007258 | JUN phosphorylation | [MAPK10, MAPK8, MAPK9] |
| GO:1900034 | Regulation of cellular response to heat | [CREBBP, EP300, MAPK3, SIRT1, YWHAE] |
| GO:1900739 | Regulation of protein insertion into mitochondrial membrane involved in apoptotic signaling pathway | [MAPK8, SFN, YWHAB, YWHAE, YWHAG, YWHAH, YWHAQ, YWHAZ] |
| GO:0030518 | Intracellular steroid hormone receptor signaling pathway | [CTNNB1, EP300, ESR1, RAN, SIRT1, YWHAH] |
| GO:0048617 | Embryonic foregut morphogenesis | [CTNNB1, SMAD2, SMAD3] |
